# Out-of-pocket payments in the Austrian healthcare system – a distributional analysis

**DOI:** 10.1186/s12939-015-0230-7

**Published:** 2015-10-14

**Authors:** Alice Sanwald, Engelbert Theurl

**Affiliations:** Department of Economics and Statistics, University of Innsbruck, Universitaetsstrasse 15, A-6020 Innsbruck, Austria

**Keywords:** Out-of-pocket health expenditure, Healthcare financing inequalities, Kakwani index, Vertical equity, Horizontal equity, Reranking, H22, H23, H51, I14

## Abstract

**Background:**

Out-of-pocket spending is an important source of healthcare financing even in countries with established prepaid financing of healthcare. However, out-of-pocket payments (OOPP) may have undesirable effects from an equity perspective. In this study, we analyse the distributive effects of OOPP in Austria based on cross-sectional information from the Austrian Household Budget Survey 2009/10.

**Methods:**

We combine evidence from disaggregated measures (concentration curve and Lorenz curve) and summary indices (Gini coefficient, Kakwani index, and Reynolds–Smolensky index) to demonstrate the distributive effects of total OOPP and their subcomponents. Thereby, we use different specifications of household ability to pay. We follow the Aronson–Johnson–Lampert approach and split the distributive effect into its three components: progressivity, horizontal equity, and reranking.

**Results:**

OOPP in Austria have regressive effects on income distribution. These regressive effects are especially pronounced for the OOPP category prescription fees and over-the-counter pharmaceuticals. Disaggregated evidence shows that the effects differ between income groups. The decomposition analysis reveals a high degree of reranking and horizontal inequity for total OOPP, and particularly, for therapeutic aids and physician services.

**Conclusions:**

The results – especially those for prescription fees and therapeutic aids – are of high relevance for the recent and on-going discussion on the reform of benefit catalogues and cost-sharing schemes in the public health insurance system in Austria.

## Background

Out-of-pocket payments (OOPP) in the healthcare sector are substantial. Roughly 40 % of the total global healthcare bill is financed from this source, and in several world regions, the OOPP share is as high as 60–70 % [1]. However, even in OECD-countries (Organisation for Economic Co-operation and Development) – mainly countries with well-established prepaid schemes of health care financing – the share of OOPP in total healthcare spending is close to 20 % (non-weighted OECD average based on the most recent information) [2]. OOPP are not only important from a fiscal perspective, they also have important consequences for economic welfare. Health expenditure is to a large extent unpredictable from an individual perspective and reduces funds to consume other necessities and amenities of life. If individuals or households were risk averse, they would demand risk-pooling mechanisms to smooth their consumption paths against the irregularities caused by bad health, in the private insurance market and/or in the political arena. Consequently, the avoidance of OOPP by pooling health expenditure risks seems to have high potential for welfare improvement. This is especially true if OOPP exceed a substantial threshold and/or push the individual or household below the poverty line [3–7]. In addition, it is well known from the previous empirical literature that major reliance on OOPP is likely to have a regressive impact on income distribution [4–9]. Finally, OOPP act as a barrier for healthcare use and might have negative effects on long-term health status, especially for low-income individuals or households [7, 10–12]. On the other hand, if administrative costs of prepaid systems are substantial and/or moral hazard exists, OOPP might improve social welfare [10].

In this study, we focus on the effects of OOPP on income distribution. This research question is embedded in the broader context of equity and fairness in the healthcare sector [3, 11, 12]. Ultimately, any analysis of whether a healthcare system fulfils the benchmarks of equity and fairness has to consider healthcare payments and service utilisation simultaneously. In this study, we focus only on healthcare payments.

We study the redistributive effects of OOPP for the Austrian healthcare system. Basically the Austrian health care system is a two tiered health care system. The public health insurance system represents the first tier. Membership in this system is obligatory not only for wage earners in the public and private sector, but also for self-employed persons (including farmers) and individuals receiving a pension from a public pension fund. Individuals with family ties to obligatory insured persons and without own public insurance coverage obtain free insurance coverage. Overall, the public health insurance system covers around 99.0 % of the whole population, excluding only marginal groups. It is mainly financed by income related contributions paid by employers and employees. These contributions are the sole source of financing public outpatient care, while public inpatient care is additionally co-financed by the states and the communities out of taxes. Private health insurance and OOPP constitute the second tier of the Austrian health care system. Roughly 35 % of the population has signed contracts with private sickness funds, which predominantly offer additional coverage to the first tier services and/or improve the possibility to choose from a broader portfolio of providers within the system. The range/level of services financed by the public health insurance system and the states is fixed in benefit catalogues agreed between providers and financing institutions. These benefit catalogues are quite comprehensive and include almost all services which are state of the art.

Outpatient health care services are supplied by physicians having a contract with the public health insurance system and by private physicians. Contracted physicians generate income from fee-for-services and lump-sum-payments. Their spatial distribution follows a capacity plan agreed between the public health insurance system and the chamber of physicians. In contrast to contracted physicians, their private counterparts are free to choose their practice location. Their remuneration is fixed in consultations between the doctor and the patient, mainly based on a fee-for-service system.

Public inpatient health care services (including outpatient services of hospitals) are supplied by public hospitals financed on a DRG-basis (Diagnosis-related-groups-basis). Private inpatient health care services are offered by private – non-profit or profit – hospitals and by private departments of public hospitals.

Patients with public health insurance coverage are basically free to consult public or private health care providers. While the utilization of public health care services is based on a benefit-in-kind-scheme with only limited cost-sharing elements, treatment costs in the private sector (i) have to be paid out-of-pocket, (ii) are born by the private sickness funds and/or (iii) by the public health insurance. The latter only finances parts of the services offered by private providers, essentially based on the benefit catalogue and the remuneration scheme for public health care institutions. So financing mode (iii) includes substantial cost sharing for the patients. Patients with private health insurance normally pay their providers directly and get their money back on agreed principles of the insurance treaty. Pharmaceuticals in inpatient care are part of the hospital remuneration within the DRG-system. In the outpatient care sector a positive list of pharmaceuticals exists which are paid by the public insurance system.

This study aims to analyze the distributive effects of OOPP in Austria. For the empirical analysis we use cross-sectional information on OOPP derived from the Austrian Household Budget Survey 2009/10 [13–15]. To measure the redistributive effects of OOPP, we apply different and complementary concepts [16–19] as follows: (i) disaggregated measures (concentration curve and Lorenz curve), (ii) summary indices (Gini coefficient, Kakwani index, and the Reynolds–Smolensky index) augmented by the Aronson–Johnson–Lampert approach [16], which splits redistributive effects into the following components: progressivity, horizontal equity, and reranking.

## Methods and data analysis

### Definitions

Any evaluation of the redistributive effect of OOPP needs a working definition of OOPP and a benchmark to classify redistributive effects [7, 11, 12]. OOPP are expenditures on healthcare services by individuals (households) in the form of payments to healthcare providers (and sometimes to prepaid plans) net of reimbursement by prepaid plans. In contrast to financial contributions to prepaid financing schemes, OOPP are connected directly with the actual utilisation of healthcare services. OOPP create a ‘quid pro quo’ relationship between payment and the utilisation of healthcare services. We are able to define three types of OOPP as follows:

- Type 1: an individual is not covered by a prepaid plan at all and has to pay the total healthcare bill directly in the case of healthcare service utilisation (breadth of the prepaid plan)
- Type 2: although a prepaid plan exists, parts of the healthcare services offered by healthcare providers are excluded and have to be paid out of pocket (depth of the prepaid plan)
- Type 3: healthcare services are included in the prepaid plan, but the costs are not financed completely by the prepaid plan (height of the prepaid plan).

Type 3 covers different forms of partial cost sharing (proportional and absolute cost sharing and public subsidies) while type 2 refers to a product- or service-related cost sharing of 100 %. As far as the Austrian case is concerned, only the OOPP types 2 and 3 are of empirical relevance in our study. Approximately 99 % of the Austrian population relies on public health insurance coverage and we do not expect that the remaining 1 % of the Austrian population without public health insurance coverage to be part of our household sample. Owing to our expectation that OOPP of types 2 and 3 have to be judged differently from a distributional perspective, we discuss the affinity of the single categories of OOPP separated in the study with the OOPP types 2 and 3 later on in the Discussion section.

Commentators on the healthcare system widely agree that every healthcare financing system has to fulfil standards of fairness [7, 11, 12]. In this context, the following three dimensions of fairness are important [3, 12]: (i) avoiding catastrophic payments by individuals or households [3], (ii) horizontal equity, and (iii) vertical equity. Our study does not consider dimension (i)^1^ [3] and concentrates on dimensions (ii) and (iii). Different forms and schedules of financial contributions may have different effects on access to healthcare services, their outcomes, and finally health status. In the following, we suppress this ‘instrumental role’ of healthcare financing as a separate issue and concentrate on the fairness of financial contributions as an intrinsic goal of the healthcare system [3, 12]. We are aware of the fact that this approach is especially controversial in the case of OOPP (see the discussion section). Thus, the rule that individuals and households should contribute to healthcare according to their ability to pay (ATP) is the starting point of our equity analysis of OOPP. Thereby, the principle of ATP includes the two dimensions of horizontal and vertical equity [3, 5, 7, 16, 17]. Horizontal equity means that individuals and/or households with an equal ATP contribute the same amount of money to the healthcare system [5]. Horizontal equity is not just a formal principle which is easy to accomplish; it also involves the definition of equality and the identification of the ‘equals’ [16–18]. Vertical equity defines a rule for the contribution of individuals and/or households with unequal ATP [16]. In the following, the principle of vertical equity is operationalised by the rule of ‘proportionality’, which means that the share of ATP contributed to the healthcare sector should be the same for all ATP levels [3].

### Statistical concepts of distributive effects

To exploit the informational content of the OOPP- and ATP-distributions efficiently, we combine disaggregated and aggregated measures to evaluate the redistributive effects of OOPP. Disaggregated evidence, which allows differentiated assessments across different ATP groups is presented graphically by comparing the Lorenz curve of ATP and the concentration curves for total OOPP and different OOPP categories [20]. Aggregated evidence is based on (i) the Gini coefficient for ATP, (ii) the Kakwani index [17] for total OOPP and OOPP categories, and (iii) the Reynolds–Smolensky index [18] for the overall redistributive effect of OOPP on the ATP distribution. Thereby, the Kakwani index *K* is defined in the following way [20]:

1 $$ K={C}_{OOPP}-{G}_{ATP} $$

where *C*_*OOPP*_ is the concentration index for total OOPP and its different subcategories and *G*_*ATP*_ is the Gini coefficient for ATP before subtracting OOPP (pre-OOPP ATP). The Reynolds–Smolensky [19] index is defined:

2 $$ \mathrm{R}\mathrm{S}=\left(\frac{t}{1-t}\right)\;K $$

where *t* is the OOPP share on sample average (OOPP/pre-OOPP ATP). Thus, the size of the Reynolds-Smolensky index measuring the redistributive effect of OOPP depends on *K* and the OOPP share. To measure ATP, we use two versions: ATP I, which represents household net income as reported by Statistik Austria, and ATP II, which adjusts for the fact that ATP should reflect free disposable household income after subtracting expenditure for the basic necessities of life (excluding OOPP). The literature [3] offers a broad discussion of adequate indicators for this expenditure. We opt to derive ATP II by subtracting the monetary value of benefits from the means-tested income maintenance program in Austria from ATP I (2010 monthly values: 744 € for a single individual; 1.108 € for a couple; 134 € for every child).^2^ Both versions of ATP and all forms of OOPP are adjusted by an equivalence scale. We use the equivalence scale provided by Statistik Austria [13, 14], in which the first adult (age >14 years) receives a value of 1, additional adults a value of 0.5, and every child (age ≤14 years) a value of 0.3.

The overall redistributive effect of OOPP on ATP – measured by the Reynolds–Smolenski index – not only depends on the vertical effect of OOPP on ATP, but also includes any horizontal inequity associated with the financing mechanism and the extent of any reranking resulting therefrom. Aronson, Johnson, and Lampert [16] worked out that we are only allowed to associate the redistributive effect of OOPP with the vertical dimension of redistribution if individuals with the same ATP pay the same contribution and the financing mechanism does not change the ordering of the ATP distribution, that is, if reranking does not exist. Aronson, Johnson, and Lampert offer a method to split redistributive effects, *RE*, into its three components, namely, vertical redistribution, *V*, horizontal inequity, *H*, and reranking, *R*. They define *RE* as:

3 $$ RE=V-H-R $$

With

$$ \begin{array}{c}\hfill V=\left(\frac{t}{1-t}\right)K\hfill \\ {}\hfill H={\displaystyle \sum {\alpha}_{ATP}\;{G_F}_{(ATP)}}\hfill \\ {}\hfill R={G_{ATP}}_{- OOPP}-{C_{ATP}}_{- OOPP}\hfill \end{array} $$

The precondition for this separation procedure is to divide the households into groups of equals in their pre-OOPP ATP. [3, 20]. We group our sample into 50 groups of pre-OOPP ATP equals by using an equal bandwidth. We split *RE* into the horizontal inequality component *H*. This captures the differences resulting from OOPP for the various groups of pre-OOPP ATP equals. Inequality in the post-OOPP ATP (ATP – OOPP) is measured in each group of pre-OOPP ATP equals by the Gini coefficient G_*F*(*ATP*)_. Using *α*_*ATP*_ as weights, a weighted sum of the Gini coefficients is calculated as the product of the household share and post-OOPP ATP share of households with a given pre-OOPP ATP [20]. *V* shows the level of ATP redistribution caused by the fact that, on average, households at different points in the ATP distribution pay different amounts of OOPP. Finally, the term *R* captures the movements of the households along the ATP distribution (reordering of the ATP distribution) in the transition from the pre-OOPP ATP distribution to the post-OOPP ATP distribution. *R* is measured by the difference between the Gini coefficient for post-OOPP ATP and the concentration index for post-OOPP ATP where, in the latter case, households are ranked by the pre-OOPP ATP [20]. The calculation of the different distributive effects was done in STATA 12.1 and closely follows the procedures proposed by O’Donnel et al. [20].

### Data

#### Data collection

To reveal the distributional effects of OOPP in Austria, we use data from the Household Budget Survey 2009/10 conducted by the National Statistical Service Office, Statistik Austria [13–15]. The observation unit is the private household without institutionalised households (hospitals, long-term care, and jail). The total sample offered by Statistics Austria consists of 6,534 households with 15,540 household members. The exclusion of 510 households with undefined or unclear household structures resulted in a final sample size of 6,024 households. Information on consumer behaviour is gathered in two ways: (i) the diary approach (observation period of 2 weeks) and (ii) the recall approach (observation period of 1 year). The recall approach is used for consumer durables and irregular/seasonal expenditure within the last 12 months. Important socio-economic characteristics of the household (e.g. family structure, age, household income) are gathered from face-to-face interviews.

#### Analysed OOPP-categories

We opt to study the redistributive effects of OOPP for total OOPP and for four important categories of OOPP. These categories are:

- *Prescription fees.* Pharmaceuticals, which are part of outpatient treatments provided by GPs/specialists who have contracts with the public health insurance system, are essentially free for the patient if they are included in the positive list of the Reimbursement Code of the public health insurance system.^3^ Each patient has to pay a prescription fee for every pharmaceutical prescribed. This prescription fee is an absolute amount of money (2009: 4.90 €; 2010: 5.00 €) with no link to the price of the pharmaceutical.^4^ Two schemes influence the financial burden of households and are, therefore, important for distributive considerations. There is an exemption from the prescription fee.^5^ Since 2008, this exemption is accompanied by a prescription fee cap at a 2 % share of annual net household income. Prescription fees of the Austrian type belong to the type 3 OOPP (see definitions). From an economic viewpoint, they amount to a specific tax per prescription, which has to be paid when consuming pharmaceuticals within a publicly provided or financed outpatient healthcare treatment.^6^
- *Over-the-counter (OTC) pharmaceuticals.* This type of OOPP occurs under two circumstances: (i) if patients rely on self-medication and (ii) if patients rely on the professional healthcare system, but the pharmaceuticals prescribed during medical treatments are not listed in the Reimbursement Code of the public health insurance and are not refunded by the private health insurance system.^7^ OTC pharmaceuticals belong to type 2 OOPP.
- *Therapeutic aids.* These include a heterogeneous package of medical resources (e.g. glasses, lenses, crowns, and bridges). When patients need therapeutic aids within publicly provided or financed healthcare services, they are confronted with different schedules of substantial cost sharing (proportional cost sharing, absolute cost sharing, and price subsidies). To a minor extent, OOPP for therapeutic aids result from utilising the private healthcare sector. In summary, consumption of therapeutic aids leads to type 2 or 3 OOPP.
- *Physician services.* This category includes physician services in the inpatient and outpatient sectors, including dental services. OOPP for physician services are either type 2 or 3. Farmers, employers, and public workers (including their relatives, this amounts to approximately 20 % of the Austrian population) face proportional cost sharing of 20 % for physician services (type 2 OOPP). If patients consume services in the private healthcare sector, they have to pay the full price. If the services are covered by public or private prepaid plans, they receive a share of the money back (type 2 or 3 OOPP).

#### Summary statistics

We demonstrate the significance of OOPP in Austria by using two indicators: (i) OOPP per capita (in PPP/US$) and (ii) OOPP as a share of total healthcare expenditure. In an international comparison, both indicators show a high degree of heterogeneity between OECD-countries [2]. The unweighted OECD average in 2012 is 560 US$ on indicator (i) and 18.5 % on indicator (ii). The values for Austria are slightly higher than the average for indicator (i) and slightly lower than the average for indicator (ii). In a time-series perspective covering the period since 1995, the share of OOPP on total healthcare expenditure in Austria is remarkably stable. OOPP account for approximately 75 % of all private health expenditure, leaving 25 % accounted for by private health insurance. In addition, we observe high stability for the share of total private consumption spend for health purposes. Based on the ESVG-1995 classification, Austrian households currently spend on average 3.5 % of total private consumption expenditure on healthcare goods and services [14].

Table 1 shows the total amount of OOPP and the OOPP for different categories based on information from the Austrian Household Budget Survey 2009/10. The first two columns show the average expenditure per month and the expenditure structure for the total sample of households. Column 3 reveals the average expenditure for households with OOPP > 0. Column 4 shows the number of households with OOPP > 0 in each expenditure category. The last column shows the expenditure structure for total health expenditure (including all sources of healthcare financing). Table 1 reveals the very specific expenditure structure of OOPP. The most important OOPP category is therapeutic aids, with 44 % of total OOPP. On the other hand, 17 % of OOPP are spent on pharmaceuticals, either directly (OTC pharmaceuticals) or indirectly, via payment of the prescription fee in the public health insurance system. Physician services (including inpatient, outpatient, and dental services) account for 26 % of OOPP. The expenditure structure of OOPP differs sharply from the structure of total health expenditure. This is especially obvious for physician services (26.55 % vs. 71 %) and therapeutic aids (44.28 % vs. 5 %). In addition, Table 1 shows the number of households with OOPP > 0 in the observed period and the average expenditure per household. As expected, we observe that the share of households without OOPP for physician services is comparably high within the observation period of 2 weeks. The comparison of the average values in columns 1 and 3 reveals the specific characteristics of the OOPP data: skewness, excess zeros, and heavy right tails.

**Table 1:** Expenditure for total OOPP and OOPP categories

Expenditure	Total households (HH)		HH with expenditure > 0		Total health expenditure
Categories	Average exp.	Percentage	Average exp.	Number of HH	Percentage
Prescription fee	6.79	6.59	34.59	1,183	−^b)^
OTC pharmaceuticals	10.88	10.55	41.08	1,596	13.00
Therapeutic aids	44.28	42.96	86.04	3,100	5.00
Physician services^a^	26.55	25.76	207.42	771	71.00
Other expenditure	14.58	14.14	54.12	1,623	11.00
Total OOPP	103.08	100.00	136.68	4,543	100.00

^a^Includes inpatient and outpatient physician services as well as dental services

^b^Prescription fees are not a health expenditure category in this expenditure classification. The source for the last column is [31]

## Results

### Results of disaggregated measures

**Fig. 1:**
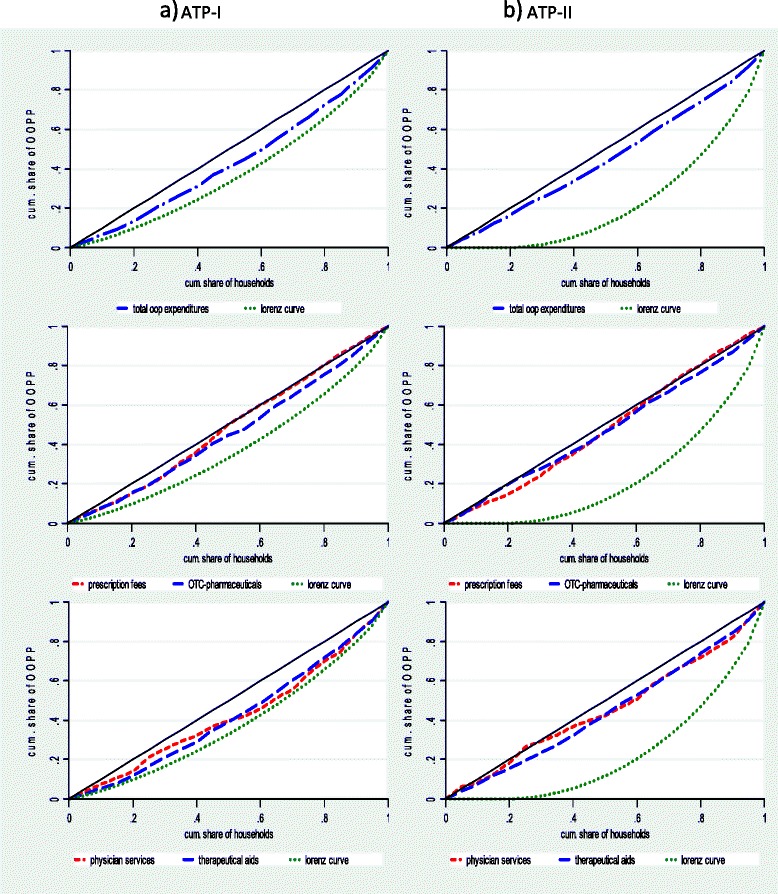
Lorenz curve of pre-OOPP ATP and concentration curves of total OOPP and OOPP categories **a**) ATP-I **b**) ATP-II

Figure 1 provides disaggregated information on the redistributive effect of total OOPP and OOPP categories by comparing the Lorenz curve of pre-OOPP ATP and the concentration curves of total OOPP and of several OOPP categories.^8^ The basis of the figures are grouped data of the households: 20 groups of equal size (each group covers 301 or 302 households) ordered by the ATP level. Every household is included with identical weight independently of its size.^9^ The left-hand side of Fig. 1 shows the Lorenz curve for ATP I , and the right-hand side shows the Lorenz curve for ATP II. We have to be aware that the two concepts of ATP do not only affect the Lorenz curve of ATP, but also the concentration curves of OOPP as they might change the ATP ranking of the households.

Related to ATP I, we observe a regressive effect of total OOPP in all ATP brackets. This regressive effect is very pronounced for prescription fees, especially in the upper ATP brackets. However, in the lower ATP parts, prescription fees also display pro-rich effects. This contrasts to the fact that there are income-related exlusions and limitations for the prescription fees. The concentration curve for OTC pharmaceuticals seems to dominate the concentration curve for prescription fees, at least in the upper parts of the ATP brackets. The regressive effects of OOPP for physician services and therapeutic aids are lower compared to OOPP for pharmaceuticals. If we relate the OOPP to ATP II, the redistributive effects are even more pronounced, especially for total OOPP and the components of physician services and therapeutic aids.

### Results of aggregated measures

Table 2 presents the distributive effects by using aggregated measures. In the upper part of the table, we present the results for ATP I, and in the lower part, we present the results for ATP II. The Gini coefficient for ATP I is 0.245.^10^ The concentration index for all OOPP categories is substantially lower compared to the Gini coefficient. This results in a regressive effect of OOPP. The negative sign of the Kakwani index confirms this statement. It is remarkable that the negative redistributive effect of the prescription fees is higher compared to OTC pharmaceuticals. Because total OOPP and its components depict only a modest share of ATP I, the overall regressive effect on ATP I measured by the Reynold–Smolensky index is moderate. The results for ATP II confirm the empirical picture for ATP I, but the regressive effect is even more pronounced, which is indicated by the remarkable increase in the Kakwani index. This is generated mainly by the doubling of the Gini coefficient. As a result, the regressive effect on ATP II measured by the Reynold–Smolensky index also increases substantially.

**Table 2:** Aggregated measures of the distributive effect of total OOPP and OOPP categories

		Total OOPP	Prescription fees	OTC pharmaceuticals	Therapeutical aids	Physician services
ATP I						
Gini coefficient	0.245	**-**	**-**	**-**	**-**	**-**
Concentration index	**-**	0.138	0.036	0.089	0.162	0.152
Kakwani index	**-**	−0.107	−0.209	−0.156	−0.083	−0.092
t = OOPP/ATP I		0.054	0.004	0.006	0.023	0.014
Reynolds–Smolensky index	**-**	−0.006	−0.001	−0.001	−0.002	−0.001
ATP II						
Gini coefficient	0.538	**-**	**-**	**-**	**-**	**-**
Concentration index	**-**	0.093	0.044	0.045	0.109	0.086
Kakwani index	**-**	−0.445	−0.494	−0.492	−0.429	−0.452
t = OOPP/ATP II	**-**	0.121	0.008	0.013	0.052	0.031
Reynolds–Smolensky index	**-**	−0.061	−0.004	−0.006	−0.024	−0.015

*ATP I* ability to pay indicated by household net income, *ATP II* ability to pay indicated by household net income minus benefits from the income maintenance program, *OOPP* out-of-pocket payment, *OTC* over the counter, *t* share of OOPP on ATP I respectively ATP II

Table 3 presents the decomposition of the redistributive effect *RE* in Austria for total OOPP and the four OOOP categories into the three components *V*, *H*, and *R* for ATP I (upper part of the table) and ATP II (lower part of the table). The results are based on ungrouped data.^11^ Thus, the coefficients and indices are slightly different compared with the grouped case (see Table 2). *RE* is captured by the Reynolds–Smolensky index. Table 3 shows the absolute values for *RE*, *V*, *H*, *R*, and the percentage decomposition of the components (baseline: *RE* = 100 %). The overall negative redistributive effect *RE* is higher in the ATP II case compared to the ATP I case. For ATP I, the negative vertical effect explains 50 % of the overall redistribution of total OOPP, leaving 17 % to horizontal inequality and 33 % to reranking. The results for the decomposition of total OOPP mask pronounced differences between the results for the different components. Approximately 90 % of the negative redistributive effect of OOPP for pharmaceuticals (prescription fees and OTC pharmaceuticals) turns out to be a distribution in favour of households with a high ATP. The values for therapeutic aids and physician services differ substantially. Horizontal inequity and reranking is much more pronounced for these OOPP categories. The high number of households without expenditure on therapeutic aids and physician services (see Table 1) and the high degree of randomness of these expenditure categories lead to a high level of reranking and horizontal inequity. For ATP II, the results differ in size, but they confirm the direction of results for ATP I.

**Table 3:** Decomposition of the redistributive impact of OOPP in Austria

	ATP	Total OOPP	Prescription fees	OTC pharmaceuticals	Therapeutical aids	Physician services
ATP I						
Gini coefficient	0.2469	**-**	**-**	**-**	**-**	**-**
RE		−0.0121	−0.0009	−0.0010	−0.0038	−0.0037
V		−0.0060	−0.0008	−0.0009	−0.0019	−0.0012
H		0.0021	0.0001	0.0001	0.0008	0.0008
R		0.0040	0.0000	0.0000	0.0011	0.0017
Decomposition (in %)						
RE (baseline)		100.00	100.00	100.00	100.00	100.00
V		0.50	0.89	0.90	0.52	0.33
H		−0.17	−0.09	−0.08	−0.21	−0.21
R		−0.33	−0.02	−0.02	−0.28	−0.46
ATP II						
Gini coefficient	0.5108	**-**	**-**	**-**	**-**	**-**
RE		−0.0770	−0.0042	−0.0067	−0.0278	−0.0211
V		−0.0595	−0.0039	−0.0061	−0.0227	−0.0142
H		0.0089	0.0003	0.0005	0.0024	0.0041
R		0.0086	0.0000	0.0001	0.0026	0.0029
Decomposition (in %)						
RE (baseline)		100.00	100.00	100.00	100.00	100.00
V		0.77	0.93	0.91	0.82	0.67
H		−0.12	−0.07	−0.07	−0.09	−0.20
R		−0.11	−0.00	−0.02	−0.09	−0.13

*ATP* ability to pay, *ATP I* ability to pay indicated by household net income, *ATP II* ability to pay indicated by household net income minus benefits from the income maintenance program, *RE* redistributive effect, *V* vertical distribution, *H* horizontal inequity, *R* reranking, *OOPP* out-of-pocket payment, *OTC* over the counter

Overall our results indicate that OOPP are regressive. This result holds for both the disaggregated and aggregated perspectives, for total OOPP, and for the different OOPP category prescription fee, OTC pharmaceuticals, therapeutic aids, and physician services. The disaggregated evidence reveals that the results differ between different income brackets. This is especially pronounced for prescription fees and physician services. The decomposition analysis reveals a high degree of reranking and horizontal inequity for total OOPP and particularly, for therapeutic aids and physician services.

## Discussion

Our study builds on previous research on the redistributive effects of state financing by taxes in general [16–19] and of different financing instruments in the healthcare sector (tax, social health insurance contribution, private health insurance premium, and OOPP) [5–9, 20–25]. The study contributes to the empirical research on the redistributive effects of OOPP in several ways. First, it takes the perspective of households and supplements the findings available at the individual level. Second, we use data from a healthcare system which is based on Bismarckian principals and which holds a specific two-tiered institutional architecture of healthcare service provision and financing. Empirical results for Austria are missing in previous (cross-national) studies, which focused on the distributional effects of different forms of healthcare financing [4–6]. Finally, we distinguish between the most important forms of OOPP and accommodate for the internal heterogeneity of this financing source. This allows us deeper insights into the distributional effects of the single components of OOPP and the overall effects.

In the following Discussion section we compare our results with previous empirical evidence and point to several open questions and limitations of our study. We also draw selected conclusions for health policy. Our results are in line with the empirical findings of the negative redistributive effect of OOPP in the previous literature. In a seminal cross-country study of 13 OECD countries, Wagstaff and Van Doorslaer find Kakwani indices for OOPP ranging from −0.037 (the Netherlands) to −0.387 (the US), with an unweighted average of the Kakwani index of −0.21 [7]. Owing to the specific income definition of ATP II, in our study, only the result for ATP I (−0.138) is comparable with the results of Wagstaff and Van Doorslaer. In a literature review, Yu, Whynes, and Sach [21] present a slightly fuller picture of 18 countries, including the results of the cross-national study of Wagstaff and Van Doorslaer [7]. They report regressive effects for the US, Switzerland, Colombia, France, Croatia, Denmark, Belgium, Portugal, Sweden, the UK, Finland, Spain, Australia, and Ireland (Kakwani index < −0.1), mildly regressive effects for Germany, Italy, and the Netherlands (Kakwani index between 0 and −0.1) and strongly progressive effects for Sri Lanka (Kakwani index: 0.548) [21]. Unfortunately, the statistical basis for this evidence is rather old. In a more recent study for Australia covering 1975–2003, Hajiuzabeh, Connelly, and Butler find Kakwani indices for OOPP in a similar range to Austria (between −0.0975 in 2003–2004 and −0.192 in 1988–1989) [9]. For Hungary, Baji, Pavlova, Gulácsi, and Groot estimate a Kakwani index for total OOPP of −0.22 in 2005–2008 [22]. For Ireland, Smith finds a regressive effect of OOPP of increasing size of the Kakwani index over 1987–2004 (1987–1988: −0.05; 1999–2000: −0.10; 2004–2005: −0.11) [24]. The empirical evidence on single OOPP categories is very scarce [24], while the used OOPP categories in [24] are hardly comparable with the classification categories used in our study. The same scarce picture applies to the decomposition of the redistributive effects into *V*, *R*, and *H*. For the Netherlands, Wagstaff and Van Doorslaer reveal components for total OOPP of the following size: *V* = 61.7 %; *H* = −11.3 %; and *R* = −27.0 % [5].

We have already mentioned several controversial issues (section 2) in our study, which are specific to the expenditure category OOPP. The concept of “equity” in healthcare financing used in our study follows the ATP approach widely favoured in the empirical literature. This approach has the advantage of allowing comparisons of OOPP with other forms of healthcare financing, particularly prepaid forms. However, this approach neglects the ‘quid pro quo’ relationship present in different categories of OOPP financing. It could be argued that redistributive considerations should take into account whether OOPP is the result of, on one hand, need-based medical treatment that is, in principle, publicly provided and financed, and which offers a standardised package of health care services (type 3 OOPP) or, on the other hand, part of the services of the private healthcare sector, which mainly complements the public healthcare sector (type 2 OOPP) on a voluntary basis. We would clearly favour the ATP approach in the first case and the fiscal equivalence approach in the second case. Unfortunately, there is no clear-cut separation between the public healthcare sector and the private healthcare sector in Austria, but tentative conclusions are possible (see our description of the Austrian health care system in section 1) . The prescription fees and therapeutic aids have a strong affinity to publicly organised medical treatment while the OTC pharmaceuticals and physician services^12^ are strongly linked with private healthcare provision. For health policy implications, it seems to be remarkable that the OOPP category prescription fee shows the highest regressive effect and that owing to the high share of OOPP for therapeutic aids (43 % of total OOPP), the negative redistributive effect – measured by the Reynold–Smolensky index – is highest. Recently, Austria’s health policy reacted to the latter case and reduced the cost sharing for therapeutic aids in dentistry.

As in other studies on the inequality of OOPP, the information on OOPP is based on survey data and, therefore, may be subject to potential bias typical of the survey method. As reported, information on consumer behaviour is gathered in two ways via the diary and recall approaches. The diary system covers 2 weeks and results in 1 year of bookkeeping, which allows the representation of seasonal patterns and specific consumption periods (e.g. waves of influenza). The recall approach is used for consumer durables and irregular or seasonal expenditure within the last 12 months. In addition, in general, households are asked for expenses greater than 300 € in the last year using the recall method. As far as our OOPP categories are concerned, only information on therapeutic aids in ophthalmology and dentistry is collected by the recall method. Overall, high data quality is ensured via the prevailing of the diary system and the level of instructions for the participants. Potential underreporting of the expenditure level of OOPP is reduced by the use of a disaggregated approach that asks for several OOPP categories.

On the other hand, the short observation period of 2 weeks used in the diary approach is a matter of concern. Shorrocks shows under quite general conditions that income inequality and the income accounting period are related negatively [26]. Owing to the specific character of health expenditure, this finding also applies at a progressive rate to the relationship of inequality in expenditure and the expenditure accounting period in the healthcare sector. The results in Table 1 are a clear indication of this finding. The consequences of the observation period are mixed. First, different acounting periods apply for the different OOPP categories and the ATP, which might influence the observed degree of inequality. Second, as far as the assessment is based on grouped data (Fig. 1 and Table 2), we rely on ATP bracket averages. Bias in this context occur primarily if these averages are sensitive to the length of the accounting period. Third, the problem is serious if the estimation of the distributive effects is based on ungrouped data. This is particularly relevant for the decomposition case into the components *V*, *R*, and *H*.

To substantiate this requires a broader discussion of the decomposition exercise for OOPP data. The decomposition method was derived first for the tax case [16]. If individual/households face the same (income) tax schedule and the tax basis exactly represents ATP, then *R* and *H* are zero. If taxation only follows ATP on average, or if taxation systematically neglects ATP, then *R* and *H* are positive, that is, violations of horizontal equity and reranking occur. This clearly means that in both the tax case and publicly prepaid schemes of healthcare financing via taxes or income-related public insurance contributions, the existence of *R* and *H* is to a high degree a matter of political design and information asymmetry between the financing institution and the payer. However, in the case of OOPP, *H* and *R* require a different interpretation. OOPP are not related systematically to ATP, and so, values of *H* and *R* that do not equal zero will be normal. They occur primarily because healthcare utilisation and, consequently, the existence and level of OOPP, are random on the individual and household level and are based on systematic risks (e.g. age, education, consumption behaviour, and family size). Only to a very limited extent are *H* and *R* for OOPP open to political design (e.g. by ATP-related limitations of cost sharing). Consequently, we would expect that *H* and *R* for OOPP are much higher compared with prepaid financing. The opposite is true for *V*. In the decomposition exercise for the Netherlands, Wagstaff and Van Doorslaer clearly confirm this expectation (*V* for direct taxation: 100.2 %, *V* for indirect taxation: 99.7 %, *V* for sickness fund contributions: 90.4 %, *V* for OOPP: 61.7 %) [5]. It is well documented that the decomposition results depend on the definition of the groups of pre-OOPP equals [25]. The decomposition exercise is based on ungrouped data. If the inequality of OOPP between households depends on the accounting period, the decomposition results would also depend on the accounting period. We would expect relatively lower values for *H* and *R* if the accounting period were extended.

Our analysis focuses on OOPP only. We find that the effect is clearly regressive. However, the share of OOPP on household income is low – on average, 3.5 % of ATP I – and so, the negative effect on the ATP distribution measured by the Reynolds–Smolenski index is limited. For several reasons, our dataset is not suitable to study the redistributive effects of other financing sources (taxation, public health insurance, and private health insurance) of the Austrian healthcare system. However, we can use recent empirical evidence from other studies to complete the picture. Guger et al. analyse the redistributive effect of public financing in Austria in general [27] and find a neutral effect of taxation on the ATP distribution. Thereby, the progressive effect of direct taxation is compensated by the regressive effect of indirect taxation. The contributions to public health insurance show a clear regressive effect. The contribution rate is proportional to income and is combined with a maximum contribution basis. In addition, the contributions to the public health insurance are tax deductible and owing to the progressive scheme of the income tax, the regressive effect increases [28]. Finally the contribution basis includes only parts of the individual ATP, mainly earned income from employers and employees, excluding capital income. This also aggravates the negative distributive impact. This additional information from other financing sources clearly shows that healthcare financing in Austria is clearly regressive overall.

## Conclusions

This study aimed to assess the redistributive effects of healthcare financing via OOPP in Austria. Disaggregated and aggregated measures were used to estimate the effect of household income distribution. The study used cross-sectional information on OOPP and income from the latest Austrian Household Budget Survey in 2009/10. The study focused on the financing side of OOPP and excluded the simultanous consideration of financing and utilisation. Our results indicate that OOPP are clearly regressive. The disaggregated evidence reveals that the results differ between different income brackets. This is especially pronounced for prescription fees and physician services. The decomposition analysis reveals a high degree of reranking and horizontal inequity. However, we should be aware that the decomposition exercise has a specific meaning in the OOPP case. The results, especially those for prescription fees and therapeutic aids, are of high relevance for recent and on-going discussions in Austria on the reform of benefit catalogues and cost-sharing schemes in the public health insurance system.

## Footnotes

1 Our calculations show that owing to the comprehensive level of public healthcare coverage, the problem of catastrophic healthcare expenditure is of low priority in Austria. The percentage of households with OOPP shares above predefined income thresholds is not related strongly to household ATP. Approximately 9 % of households exceed the 2 % threshold, 4.3 % exceed the 5 % threshold, and 1.6 % exceed the 10 % threshold. We expect this percentages to decrease if the accounting period is increased.

2 Households which have a negative ATP II are given an ATP II of zero in the calculations.

3 Pharmaceuticals consumed within inpatient treatment are free for the patient. Their financing is included in the DRG-based hospital financing system, and there is no prescription fee.

4 If the price of the pharmaceutical is below the prescription fee, the patients pay the price of the pharmaceutical.

5 Exemptions are granted without application for (a) retired people who draw small pensions from public pension plans, (b) people with notifiable communicable diseases, (c) members of the civilian service, including their relatives, and (d) asylum seekers. On application, exemptions from the prescription fees are granted for insurance members (including co-insured household members), each with a household net income below the threshold values of the basic income maintenance system.

6 If patients consume outpatient medical services supplied by private physicians, pharmaceuticals are paid by the public health insurance system on request, and the system of prescription fees is applied in a similar way.

7 Expenditure from OTC pharmaceuticals can result from publicly or privately provided medical treatment. Private health insurance plays only a very limited role in financing pharmaceuticals.

8 We abstain from showing the redistributive effects of the OOPP category “other expenditure” because it includes a heterogeneous mix of different expenditure items.

9 Thereby, we are aware that in distributional matters, the individual rather than the household is regarded as the relevant unit and, correspondingly, individuals have to be ranked in ascending order according to their household ATP. This is analogous to weighting the household ATP by the number of household members [29, 30].

10 This result is in line with those of comparable studies on the income distribution in Austria [27]

11 Ungrouped data are needed for the decomposition of *RE* into *V*, *R*, and *H* [20].

12 The proportional cost sharing of members of the health insurance for public workers, employers, and farmers is excluded in this assessment.
